# Delta Sequential Organ Failure Assessment Within the First 24 Hours as a Predictor of Mortality in Critically Ill Surgical Patients: A Retrospective Study

**DOI:** 10.7759/cureus.109229

**Published:** 2026-05-19

**Authors:** Richa Tiwari, Rahul Chauhan, Nupur B Patel

**Affiliations:** 1 Critical Care Medicine, Shri Guru Ram Rai Institute of Medical and Health Sciences, Dehradun, IND

**Keywords:** critically ill, delta sofa, mortality, multiple organ dysfunction, sofa score, surgical icu

## Abstract

Introduction: Early identification of critically ill surgical patients at high risk of mortality is essential for timely intervention. The Sequential Organ Failure Assessment (SOFA) score is widely used to assess organ dysfunction, and dynamic changes in SOFA may provide better prognostic value than a single measurement. This study aimed to evaluate the role of change in SOFA score within the first 24 hours (ΔSOFA) as a predictor of mortality in surgically critically ill patients with shock and multiple organ dysfunction syndrome (MODS).

Methods: This retrospective observational study was conducted in a tertiary care intensive care unit (ICU). Adult surgical patients (≥18 years) with shock and MODS were included. SOFA scores were calculated at admission and at 24 hours. ΔSOFA was determined as the difference between these values, and patients were categorized into ΔSOFA 2-4 and ΔSOFA >4 groups. The primary outcome was in-hospital mortality. Statistical analysis was performed using Fisher’s exact test, with p < 0.05 considered significant.

Results: A total of 200 patients were included. The overall mortality rate was 40%. Among patients with ΔSOFA 2-4 (n = 68), mortality was 58.8%, whereas patients with ΔSOFA >4 (n = 34) had a significantly higher mortality of 94.1% (p = 0.002). Patients with ΔSOFA >4 had markedly higher odds of mortality compared to those with ΔSOFA 2-4 (odds ratio (OR) = 11.2, 95% confidence interval (CI): 2.5-50.8). Most patients had severe disease at admission, with high baseline SOFA scores and multi-organ involvement. Infectious etiology was the predominant cause of MODS.

Conclusion: An increase in SOFA score within the first 24 hours is strongly associated with mortality in surgically critically ill patients. ΔSOFA is a simple, objective, and dynamic tool that can aid in early risk stratification and guide clinical decision-making in the ICU.

## Introduction

Critically ill surgical patients admitted to the intensive care unit (ICU) often present with severe physiological derangements due to trauma, major surgery, sepsis, hemorrhage, or postoperative complications. These conditions frequently lead to circulatory shock and progressive organ dysfunction, which are major contributors to mortality. Early identification of high-risk patients is essential for timely intervention and improved outcomes.

Multiple Organ Dysfunction Syndrome (MODS) commonly develops in such patients due to systemic inflammation and persistent tissue hypoperfusion, significantly increasing mortality risk [1]. Therefore, objective tools for assessing organ dysfunction and predicting outcomes are crucial in their management.

The Sequential Organ Failure Assessment (SOFA) score is widely used to quantify organ dysfunction in critically ill patients [2,3]. It evaluates six organ systems, i.e., respiratory, cardiovascular, hepatic, renal, neurological, and coagulation, using routine clinical and laboratory parameters. Higher SOFA scores are associated with increased mortality.

While the admission SOFA score reflects initial disease severity, serial assessment provides better insight into patient progression. Changes in SOFA score during the first 24 hours (ΔSOFA) have been shown to correlate with outcomes [4], with increasing scores indicating worsening organ dysfunction and higher mortality, and decreasing scores suggesting recovery.

While the Sepsis-3 definition identifies ΔSOFA ≥2 as clinically significant, it does not differentiate the prognostic impact of varying magnitudes of change. Organ failure progression is nonlinear, with ΔSOFA 2-4 reflecting early, potentially reversible dysfunction, and >4 indicating advanced multi-organ failure with markedly higher mortality. Thus, stratifying ΔSOFA extends SOFA from a diagnostic tool to a dynamic risk assessment model.

In surgically critically ill patients with shock and MODS, early dynamic assessment using SOFA may help identify those at higher risk [5]. However, data on the prognostic value of early SOFA changes in this population remain limited.

This study was conducted to evaluate the role of 24-hour change in SOFA score as a predictor of mortality in surgically critically ill patients with shock and MODS.

## Materials and methods

Study design and population

This retrospective observational study was conducted in the Department of Critical Care Medicine ICU at Shri Guru Ram Rai Institute of Medical and Health Sciences and the associated Shri Mahant Indiresh Hospital, Dehradun, India. The study was conducted over 18 months, from June 1, 2024, to December 31, 2025, and included surgically ill patients. Adult patients (≥18 years) admitted with shock and MODS were evaluated. Shock was defined as persistent hypotension (mean arterial pressure <65 mmHg) requiring vasopressor support despite adequate fluid resuscitation, and MODS as dysfunction of two or more organ systems.

Exclusion criteria

Patients with an ICU stay of less than 24 hours, incomplete data for SOFA score calculation, discharge against medical advice, or readmission during the same hospital stay were excluded.

Rationale of the study

Early risk stratification in critically ill surgical patients with shock and MODS is challenging due to the dynamic evolution of organ dysfunction. While the Sepsis-3 definition identifies ΔSOFA ≥2 as clinically significant, it does not differentiate the prognostic impact of varying magnitudes of change. Organ failure progression is nonlinear, with ΔSOFA 2-4 reflecting early, potentially reversible dysfunction and >4 indicating advanced multi-organ failure with markedly higher mortality. Thus, stratifying ΔSOFA extends SOFA from a diagnostic tool to a dynamic risk assessment model. This study evaluates the prognostic significance of 24-hour ΔSOFA stratification in surgically critically ill patients.

SOFA score assessment

SOFA score was calculated at ICU admission (0 hours) and at 24 hours based on six organ systems: respiratory (PaO₂/FiO₂ ratio), cardiovascular (mean arterial pressure and vasopressor requirement), hepatic (serum bilirubin), renal (serum creatinine and urine output), neurological (Glasgow Coma Scale), and coagulation (platelet count). Each organ system was scored from 0 to 4, with higher scores indicating greater severity of organ dysfunction. The total SOFA score ranged from 0 to 24. ΔSOFA was calculated as the difference between the 24-hour SOFA score and the admission SOFA score. Patients were categorized into ΔSOFA 2-4 and ΔSOFA >4 groups to differentiate between moderate progression and severe, rapidly worsening organ dysfunction.

Outcome and statistical analysis

The primary outcome was in-hospital mortality. Data were analyzed using IBM SPSS Statistics for Windows, Version 20 (Released 2011; IBM Corp., Armonk, New York). Categorical variables were compared using Fisher’s exact test. The strength of association was expressed as an odds ratio (OR) with a 95% confidence interval. A p-value <0.05 was considered statistically significant.

## Results

A total of 200 surgically ill patients admitted to the ICU with shock and multiple organ dysfunction were included in the study.

Demographic and clinical profile

The majority of patients were elderly, with 60% aged ≥49 years, and the highest proportion in the 69-88 years group (32%) (Table 1).

**Table 1:** Age distribution of patients with MODS (total number of patients = 200) MODS: multiple organ dysfunction syndrome.

Age Group (Years)	Number of Patients (%)
18-28	20 (10.0%)
29-48	50 (25.0%)
49-68	56 (28.0%)
69-88	64 (32.0%)
≥89	10 (5.0%)

There was a male predominance, with 61% male and 39% female (Table 2).

**Table 2:** Gender distribution (total number of patients = 200)

Gender	Number of Patients (%)
Male	122 (61.0%)
Female	78 (39.0%)

Infectious etiology was the most common cause of MODS, observed in 87% of patients, predominantly of abdominal origin (77%) (Table 3).

**Table 3:** Etiology of MODS (total number of patients = 200) MODS: multiple organ dysfunction syndrome.

Etiology	Number of Patients (%)
Infectious (total)	174 (87%)
Infectious (abdominal)	154 (77%)
Infectious (soft tissue)	20 (10%)
Traumatic	18 (9.0%)
Pancreatitis	8 (4.0%)

Most patients had severe disease at presentation, with 58% involving ≥4 organ systems (Table 4).

**Table 4:** Number of organ systems involved (total number of patients = 200)

Organs Involved	Number of Patients (%)
2	24 (12.0%)
3	60 (30.0%)
≥4	116 (58.0%)

Among organ systems, respiratory involvement was most common (97%), followed by renal (80%) and cardiovascular (69%) (Table 5).

**Table 5:** Organ systems involved (total number of patients = 200)

Organ System	Number of Patients (%)
Respiratory	194 (97.0%)
Cardiovascular	138 (69.0%)
Renal	160 (80.0%)
Hepatic	102 (51.0%)
Haematological	122 (61.0%)
Neurological	80 (40.0%)

Acute kidney injury was the most frequent complication, occurring in 82% of patients (Table 6).

**Table 6:** Complications during treatment (total number of patients = 200) ARDS: acute respiratory distress syndrome, DIC: disseminated intravascular coagulation.

Complication	Number of Patients (%)
Acute kidney injury	164 (82.0%)
ARDS	44 (22.0%)
DIC	14 (7.0%)

SOFA score distribution

At admission, 54% of patients had a SOFA score ≥9, with 11% having scores ≥14, indicating severe organ dysfunction (Table 7).

**Table 7:** SOFA score at admission (total number of patients = 200) SOFA: Sequential Organ Failure Assessment.

SOFA Score	Number of Patients (%)
3-8	92 (46.0%)
9-13	86 (43.0%)
≥14	22 (11.0%)

ΔSOFA and mortality

A significant association was observed between the change in SOFA score within the first 24 hours and mortality (Table 8).

**Table 8:** Association between ΔSOFA and mortality (total number of patients =200) Statistical test used: Fisher's exact test. ΔSOFA: change in Sequential Organ Failure Assessment score within the first 24 hours.

ΔSOFA Category	Survived (n, %)	Deceased (n, %)	Total (n)	Statistical Test
ΔSOFA 2-4	28 (41.2%)	40 (58.8%)	68	p = 0.002, odds ratio (OR): 11.2, 95% CI: 2.5–50.8
ΔSOFA >4	2 (5.9%)	32 (94.1%)	34	
Total	30	72	102	

Among patients with ΔSOFA 2-4 (n = 68), 40 patients (58.8%) died, while 28 (41.2%) survived. In contrast, among patients with ΔSOFA >4 (n = 34), 32 patients (94.1%) died, with only 2 survivors (5.9%). This difference was statistically significant (p = 0.002). Patients with ΔSOFA >4 had significantly higher odds of mortality compared to those with ΔSOFA 2-4 (odds ratio (OR) = 11.2, 95% confidence interval (CI): 2.5-50.8).

Overall outcomes

The overall mortality in the study population was 40%, with 60% survival (Table 9).

**Table 9:** Survival outcomes (total number of patients = 200)

Outcome	Number of Patients (%)
Survived	120 (60.0%)
Deceased	80 (40.0%)

The mean recovery time among survivors was 7.7 ± 4.5 days. Mechanical ventilation was required in 54% of patients, and vasopressors were used in 70% (Table 10), reflecting the severity of illness.

**Table 10:** Pharmacological interventions and supportive measures (total number of patients = 200)

Intervention	Number of Patients (%)
Broad-spectrum antibiotics	190 (95.0%)
Vasopressors	140 (70.0%)
Mechanical ventilation	108 (54.0%)
Renal replacement therapy	8 (4.0%)

## Discussion

The present study evaluated the prognostic significance of early changes in the SOFA score in surgically critically ill patients with shock and multiple organ dysfunction. The findings demonstrate that an increase in SOFA score within the first 24 hours is strongly associated with mortality, highlighting the value of dynamic organ dysfunction assessment in this population.

In our study, the overall mortality was 40%, which is comparable to previously reported mortality rates in patients with MODS and sepsis, ranging between 30% and 60% depending on severity and population studied [1,6]. The majority of patients were older than 49 years, and increasing age has been consistently associated with poorer outcomes due to reduced physiological reserve and higher susceptibility to organ failure [6].

Infectious etiology was the predominant cause of MODS in our cohort (87%), particularly intra-abdominal infections [7]. This finding is consistent with existing literature, where sepsis remains the leading precipitating factor for organ dysfunction in ICU settings [6]. The high prevalence of respiratory (97%) and renal (80%) involvement reflects typical patterns observed in critically ill patients [8], with acute kidney injury being one of the most common complications and an independent predictor of mortality [9].

At admission, more than half of the patients had a SOFA score ≥9, indicating significant organ dysfunction at presentation. However, the baseline SOFA score alone may not adequately reflect disease trajectory. Several studies have emphasized that serial assessment of the SOFA score provides superior prognostic information compared to a single measurement [4,10].

The key finding of our study is the strong association between the 24-hour change in SOFA score (ΔSOFA) and mortality. Patients with a ΔSOFA increase of 2-4 had a mortality rate of 58.8%, whereas those with ΔSOFA >4 had an extremely high mortality rate of 94.1%, which was statistically significant (p ≈ 0.0021). This association was further supported by a markedly increased risk of death in the ΔSOFA >4 group (odds ratio (OR) = 11.2, 95% confidence interval (CI): 2.5-50.8). This is in line with the findings of Ferreira et al., who demonstrated that increasing SOFA scores during the initial ICU stay are closely associated with higher mortality [4]. Similarly, Vincent et al. reported that worsening organ dysfunction, as reflected by rising SOFA scores, is a strong predictor of poor outcomes [11].

The markedly higher mortality observed in patients with ΔSOFA >4 underscores the importance of early identification of rapidly deteriorating patients [12]. A rising SOFA score likely reflects ongoing tissue hypoperfusion, persistent inflammation, and failure of therapeutic interventions [13]. Therefore, ΔSOFA serves as a dynamic marker of disease progression and may help guide early escalation of care, including aggressive hemodynamic support and timely source control.

The requirement for organ support in our study population further reflects disease severity, with 54% requiring mechanical ventilation and 70% requiring vasopressors. These findings are consistent with prior studies, where the need for organ support correlates with higher SOFA scores and worse outcomes [14].

In conclusion, the present study demonstrates that early change in SOFA score within the first 24 hours is a strong predictor of mortality in surgically critically ill patients with shock and MODS. ΔSOFA is a simple, objective, and dynamic tool that can aid in early risk stratification and guide clinical decision-making in the ICU setting.

Limitations

This study has several limitations. First, it is a retrospective, single-center study, which may limit the generalizability of the findings and is subject to selection and information bias. Second, patients with an ICU stay of less than 24 hours were excluded, which may have led to underrepresentation of early mortality cases. Third, the study primarily focused on patients with increasing SOFA scores and did not include a comparison group with stable or decreasing SOFA scores, which could have provided a more comprehensive assessment of prognostic value and may have introduced selection bias. Additionally, potential confounding factors, such as variations in treatment protocols, timing of interventions, and severity of underlying surgical conditions, were not controlled. Furthermore, multivariable analysis was not performed due to the retrospective design and relatively limited sample size. Finally, the relatively small sample size may limit the statistical power of the study.

## Conclusions

The present study demonstrates that a change in SOFA score within the first 24 hours is a significant predictor of mortality in surgically critically ill patients with shock and multiple organ dysfunction. Patients with a greater increase in SOFA score, particularly those with ΔSOFA >4, had markedly higher mortality compared to those with moderate increases. These findings highlight the importance of early dynamic assessment of organ dysfunction. ΔSOFA is a simple, objective, and clinically useful tool that can aid in early risk stratification and guide timely therapeutic interventions in the intensive care setting.

## References

[1] Marshall JC, Cook DJ, Christou NV, Bernard GR, Sprung CL, Sibbald WJ (1995). Multiple organ dysfunction score: a reliable descriptor of a complex clinical outcome. Crit Care Med.

[2] Vincent JL, de Mendonça A, Cantraine F (1998). Use of the SOFA score to assess the incidence of organ dysfunction/failure in intensive care units: results of a multicenter, prospective study. Working group on "sepsis-related problems" of the European Society of Intensive Care Medicine. Crit Care Med.

[3] Ranzani OT, Singer M, Salluh JI (2025). Development and validation of the Sequential Organ Failure Assessment (SOFA)-2 score. JAMA.

[4] Ferreira FL, Bota DP, Bross A, Mélot C, Vincent JL (2001). Serial evaluation of the SOFA score to predict outcome in critically ill patients. JAMA.

[5] Kumar M, S CC (2018). SOFA scoring system in assessing prognosis of critically ill surgical and trauma patients: a prospective study. Int Surg J.

[6] Singer M, Deutschman CS, Seymour CW (2016). The Third International Consensus Definitions for sepsis and septic shock (Sepsis-3). JAMA.

[7] Sartelli M (2010). A focus on intra-abdominal infections. World J Emerg Surg.

[8] Pickkers P, Darmon M, Hoste E (2021). Acute kidney injury in the critically ill: an updated review on pathophysiology and management. Intensive Care Med.

[9] Girling BJ, Channon SW, Haines RW, Prowle JR (2020). Acute kidney injury and adverse outcomes of critical illness: correlation or causation?. Clin Kidney J.

[10] Tee YS, Fang HY, Kuo IM, Lin YS, Huang SF, Yu MC (2018). Serial evaluation of the SOFA score is reliable for predicting mortality in acute severe pancreatitis. Medicine (Baltimore).

[11] Premkumar K, Naik RR, Indurekha P, Shankar GVP, Sab MR, Dara C (2025). Battle of the scores: SOFA v/s q SOFA in forecasting critical care mortality. Eur J Cardiovasc Med.

[12] Sala-Trull MC, Monedero P, Guillen-Grima F, Leon-Sanz P (2025). Mortality predictors for ICU end-of-life decisions: delta-SOFA and SAPS 3 - retrospective evaluation. BMJ Support Palliat Care.

[13] Balle GN, Biradar SM, Kadagud AM (2026). Evaluation of the efficacy of serum lactate-to-albumin ratio as a prognostic marker for sepsis and its comparison with the Sequential Organ Failure Assessment (SOFA) score. Cureus.

[14] Pantis C, Popoviciu MS, Ghitea TC, Pop MA, Brata RD (2026). Organ support requirements as markers of disease severity and mortality in hospitalized patients. J Clin Med.

